# Anatomical Variation of the Olfactory Fossa According to Keros and Yenigun Classifications in Karachi, Pakistan

**DOI:** 10.7759/cureus.73314

**Published:** 2024-11-09

**Authors:** Muhammad Yousuf, Afifa Jamil, Qasim Hashmi, Kahkashan Hameed, Muhammad Sami, Taha Shaikh, Sadaf Zia

**Affiliations:** 1 Acute Medicine, Kingston Hospital NHS Foundation Trust, London, GBR; 2 Otolaryngology and Head and Neck Surgery, Garrison Hospital and Diagnostic Center, Karachi, PAK; 3 Ear, Nose, and Throat (ENT), Dr. Ruth K. M. Pfau Civil Hospital Karachi, Karachi, PAK; 4 Otolaryngology and Head and Neck Surgery, Hamdard Medical and Dental College, Karachi, PAK; 5 Medicine, Aga Khan University Medical College, Karachi, PAK; 6 Ear, Nose, and Throat (ENT), Dow University of Health Sciences, Karachi, PAK

**Keywords:** anatomical variation of olfactory fossa, different variation of olfactory fossa, keros and yenigun classification, olfactory fossa variation, olfactory fossa variation according to keros and yenigun classification

## Abstract

Aim: The goal of our research is to assess the olfactory fossa anatomical variation through Keros and Yenigun classification by computed tomography (CT) scan of paranasal sinuses (PNS), as in order for surgeons to prevent major complications, they must be familiar with its variations in anatomy during functional endoscopic sinus surgery (FESS) related to anterior ethmoidal artery injury, as its course varies with depth and length of the olfactory fossa.

Methods: At Karachi's Dow University of Health Sciences (DUHS), retrospective research was carried out. From anterior to posterior, sequential coronal and axial pictures were examined, and the existence and location of further anatomical characteristics were documented. A consultant radiologist used the picture archiving and communication system (PACS) software to do the review. The depth of the olfactory fossa and anterior-posterior length were measured. We utilized OpenEpi version 3 to compute the sample size findings. The estimated sample size was calculated as 249 by taking the prevalence of 63.5% for type 2 Keros classification, with a margin of error of 6% and a confidence level.

Results: The median length for the olfactory fossa depth (Keros) for the left and right sides were 6.2 (2.2-10.9) mm and 6.0 (2.8-10.4) mm, respectively. The median anteroposterior length of the cribriform plate (Yenigun) for the left and right sides were 11.4 (6.1-19.2) mm and 11.2 (6.0-18.0) mm, respectively. According to Keros Classification, type 2 was observed as the most prevalent category on the right side for both females and males, with 97/122 (79.5%) and 106/127 (83.5%) individuals, respectively. The chi-square test was applied for the analysis of gender and classification. Keros type 2 was observed to be the most prevalent in both males and females. Similarly, type 2 was also seen to be the most prevalent in the Yenigun classification in both genders. No statistical difference was found in both classifications (p = 0.39).

In most of the literature, type 2 has been the most commonly dominated type. The reason seems due to the variation in genes and anatomy of the human PNS.

Conclusion: Preoperative planning should take the morphological characteristics of the ethmoidal roof into account. Understanding the ethmoidal roof morphology among populations may be improved by looking at bigger patient groups and meta-analyses that compile different study findings on this topic.

## Introduction

More than 31 million people are affected by chronic rhinosinusitis (CRS) each year, which requires surgical attention [1]. The conventional surgical approach for treating CRS with nasal polyps is functional endoscopic sinus surgery (FESS) [2,3]. To avoid major surgical complications and difficulties, in-depth anatomical knowledge is deemed essential, particularly with regard to the skull base [4].

The cribriform plate of the ethmoid bone forms the floor of the olfactory fossa, which is a depression in the anterior cranial cavity. The anterior cranial fossa and nasal cavity are separated from each other by this thin plate of bone called a cribriform plate. The medial and lateral walls of the olfactory fossa are formed by crista galli and lateral lamella, respectively [5].

The frontal sinus in the anterior skull may be easily recognized by using the anterior ethmoidal artery (AEA) as a crucial anatomical landmark [6]. Routes of exit of AEA through the skull base are ethmoid sinus, through the bottom of the base of the skull, or it can dangle freely underneath the skull base. These routes of AEA exit are crucial, as it may be injured during FESS when it hangs below the base of the skull [7].

The anterior ethmoid artery is a crucial anatomical structure that might be accidentally damaged during FESS since it passes through the orbit and anterior ethmoidal sinus before entering into the anterior cranial fossa. The issues caused by its injury are grievous epistaxis, intraorbital bleeding, and hematoma behind the orbit, which could cause blindness, cerebral hemorrhage, and cerebrospinal fluid leakage [8].

Before going for FESS, a computed tomography (CT) scan of the nose and paranasal sinuses (PNS) is helpful for the evaluation of anterior ethmoid artery pathway, ethmoidal fovea asymmetry, and structural variances of the lateral lamella and olfactory fossa, as they are critical in FESS to prevent the iatrogenic damage [9]. Using a CT scan of the nose and PNS preceding FESS, radiologists can predictably spot anatomical variations that put patients at risk of severe surgical complications [10].

Keros assorted the olfactory fossa into three categories based on the length of the lateral lamella of the cribriform plate of the ethmoid bone in 1962 and evaluated the induced risk while doing surgical interventions in the ethmoidal area. The olfactory fossa is classified as type-1 (<3.0 mm), type-2 (4.0-7.0 mm), and type-3 (8.0-16.0 mm).

The likelihood that the AEA will run conspicuously inside the ethmoidal sinus increases, in accordance with Keros categorization, with increasing superior-inferior depth of the cribriform plate.

Yenigun in 2016 defined the "Yenigun classification." This classification attempts to locate the ethmoid sulcus in the axial section and classifies it according to the anterior-posterior length of the cribriform plate into type 1 (6-10 mm), type 2 (11-15 mm), and type 3 (16-20 mm).

There is a greater chance that the AEA will run clearly inside the sinus as the anterior-posterior length of the cribriform plate increases.

The goal of our study is to gauge the Keros types and their frequency by assessing the olfactory fossa depth corresponding to the Keros classification on CT scan PNS, as it provides the factual assessment of the anatomy of the anterior skull base and supports the surgeon in guiding during FESS to raise the patient’s safety standards. It will help surgeons anticipate the complications that may be encountered during surgery.

## Materials and methods

At Dow University of Health Sciences (DUHS), Karachi, retrospective research was carried out after receiving acquiescence from DUHS’ Institutional Review Board (IRB-2994/DUHS/Approval/2023/227).

Our inclusion criteria comprised all patients who went for a CT scan for paranasal sinus plane at the hospital with ages 18 or above. Any patients who were under 18, pregnant, underwent previous surgery for PNS and skull base, or had any congenital sinus anomaly were excluded from the study.

Paranasal sinus CT scans taken in 2 mm sections were evaluated for all patients. The CT scans are performed on patients as a routine investigation, presenting to the outpatient department of ENT in Dr. Ruth K. M. Pfau Civil Hospital Karachi with a complaint of CRS or any mass in the nasal cavity planned for endoscopic sinus surgery (FESS). A Dual Energy SIEMENS Definition Edge CT Scanner (Siemens Healthineers, Germany) was used. To identify the existence and location of additional anatomical characteristics, sequential coronal and axial pictures were examined from anterior to posterior. A consultant radiologist used the picture archiving and communication system (PACS) software to do the review. The depth of the olfactory fossa and anterior-posterior length were measured.

Using OpenEpi version 3, we computed the sample size outcomes. The approximated sample size was calculated as 249 by taking the prevalence of 63.5% for type 2 Keros classification, with a margin of error of 6% and confidence level.

IBM SPSS Statistics for Windows, Version 23 (Released 2015; IBM Corp., Armonk, New York, United States) was used to assemble and analyze the patient's data. Descriptive analyses of categorical data were provided in terms of frequencies and percentages, whereas if the data were regularly distributed, the continuous data were reported as mean ± standard deviation; if not, the data were expressed as median (range). The chi-square test was used to analyze the relationship between the gender and types of Keros and Yenigun classification. Using the Shapiro-Wilk test, normality tests were conducted. The comparisons between males and females for olfactory furrow depth and anteroposterior length of the cribriform plate for the right and left sides were made by using the two-tailed Mann-Whitney U test (nonparametric data). For statistical significance, a p-value of less than 0.05 was used.

## Results

An aggregate of 249 paranasal sinus CT images were interpreted to observe the ethmoidal sinus roof. There were 127 (51.0%) male and 122 (49.0%) female. The median age of participants was 33 (18-50) years.

The median length for the olfactory fossa depth (Keros) for the left and right sides were 6.2 (2.2-10.9) mm and 6.0 (2.8-10.4) mm, respectively. The median anteroposterior length of the cribriform plate (Yenigun) for the left and right sides were 11.4 (6.1-19.2) mm and 11.2 (6.0-18.0) mm, respectively.

**Figure 1:**
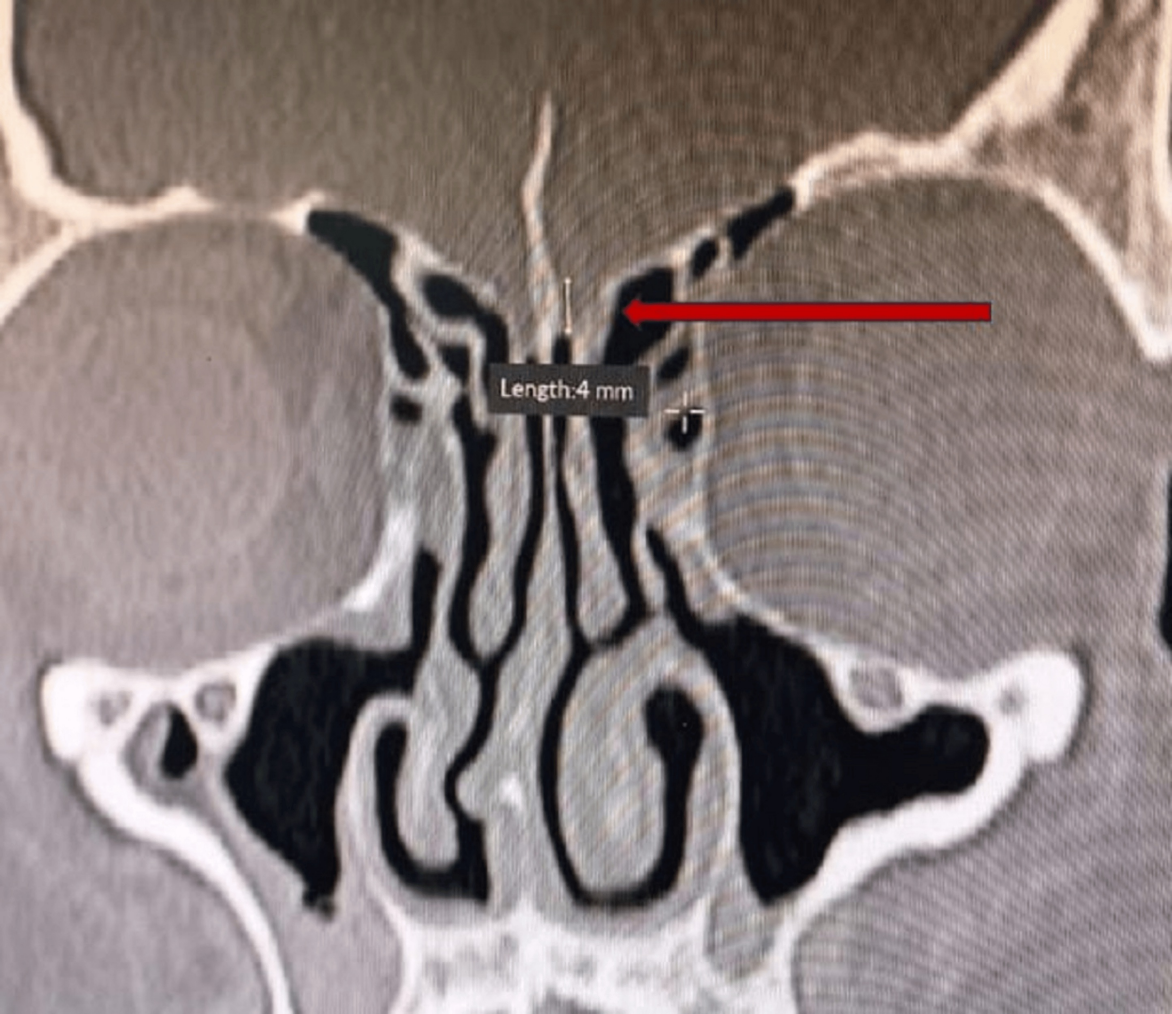
Coronal section of CT scan paranasal sinuses showing left sided type 2 Keros

**Figure 2:**
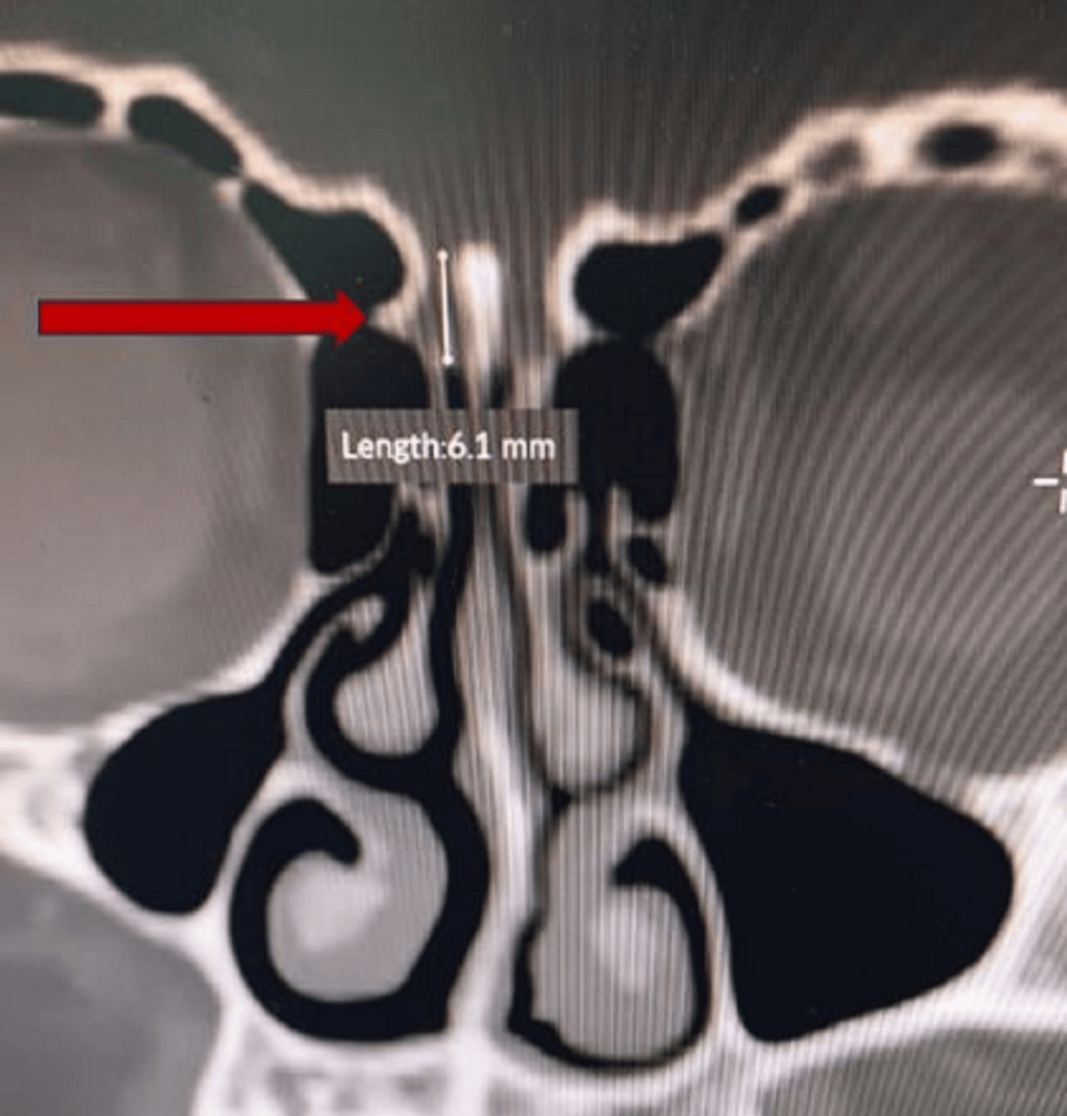
Coronal section of CT scan paranasal sinuses showing right sided type 2 Keros

According to Keros Classification, on the right side, type 2 was found to be the most prevalent type for both females and males, with 97/122 (79.5%) and 106/127 (83.5%) individuals, respectively. There was no discernible statistically significant difference between the sexes in this classification (p = 0.39). Similarly, type 2 was also the most common variation on the left side for both females and males, with 98/122 (80.3%) and 114/127 (89.8%) individuals, respectively. There was no statistically significant difference observed between males and females in this classification (p = 0.056). The left and right side measurements are shown in Figure 1 and Figure 2, respectively.

Similar to the Keros classification, type 2 was seen as the most common variation according to the Yenigun classification on both sides. About 139/249 (55.8%) individuals had type 2 class on the left side, while 133/249 (53.4%) individuals had type 2 on the right side. Unlike the Keros classification, type 1 was the second most common category, with 93/249 (37.3%) and 98/249 (39.4%) individuals for the left and right sides, respectively.

On cross-tabulation between males and females, type 2 was found to be the most common class. About 79/127 (62.2%) males and 60/122 (49.2%) females had type 2 on the left side, and 75/127 (59.1%) males and 58/122 (47.5%) females had type 2 on the right side.

**Figure 3:**
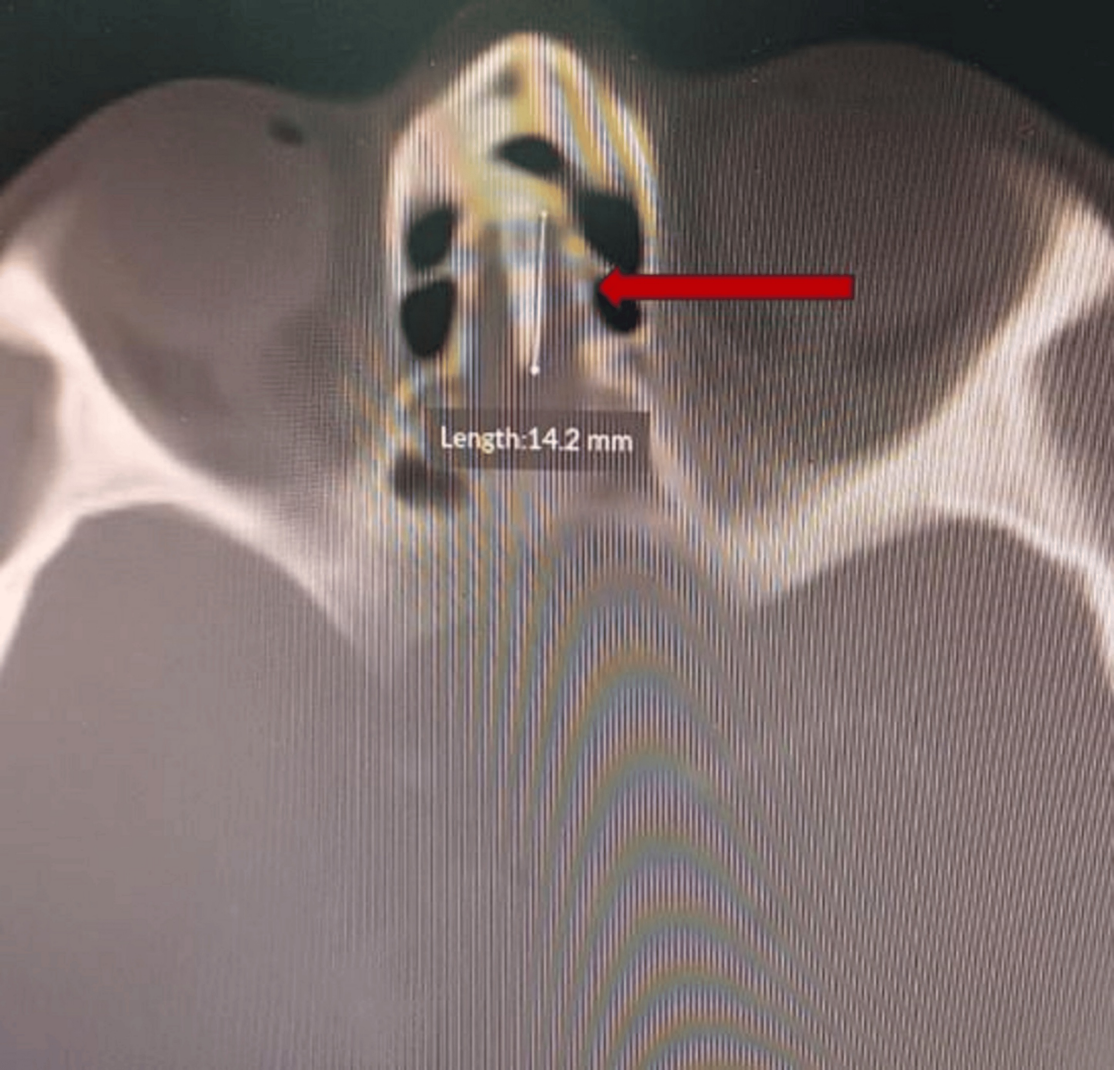
Axial section of CT scan paranasal sinuses showing left sided type 2 Yenigun

**Figure 4:**
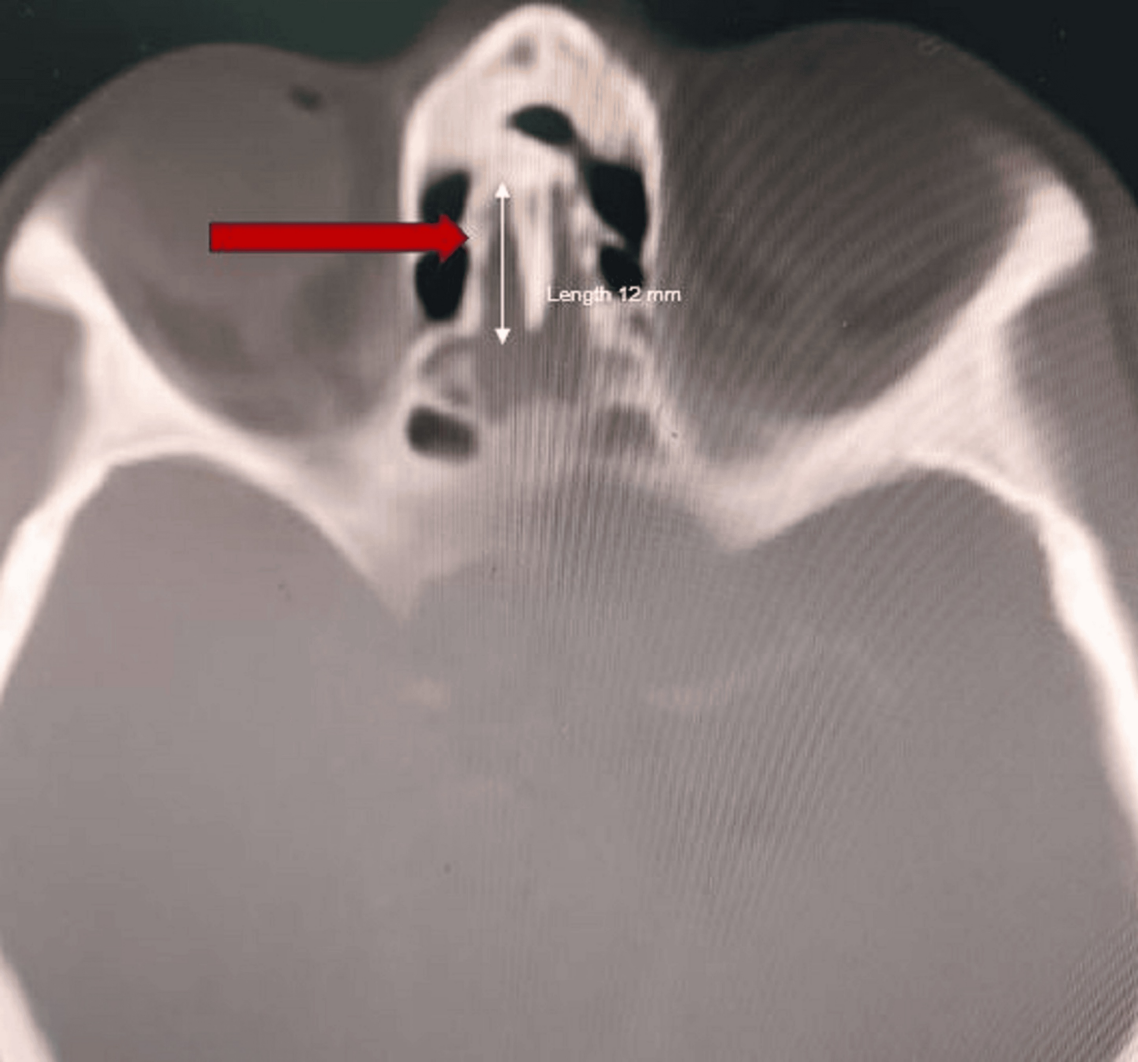
Axial section of CT scan paranasal sinuses showing right sided type 2 Yenigun

There was no significant difference observed on either the left side (p = 0.093); as shown in Figure 3, or the right side (p = 0.20); as shown in Figure 4, in this classification between males and females.

The Mann-Whitney U test did not find any differences that were statistically significant in the right anteroposterior length of the cribriform plate between males (median = 11.3, IQR = 3.6) and females (median = 11.1, IQR = 3.6) (U = 7187, p = 0.32). Similarly, no significant difference was observed for the left anteroposterior length between males (median = 11.6, IQR = 4.0) and females (median = 11.3, IQR = 4.1) (U = 7130, p = 0.28). Furthermore, the analysis indicated no significant difference in the right olfactory furrow depth (Keros method) between males (median = 5.7, IQR = 1.7) and females (median = 6.0, IQR = 2.0), U = 7513, p = 0.68, nor in the left olfactory furrow depth between males (median = 6.2, IQR = 1.7) and females (median = 6.3, IQR = 1.8) (U = 7426, p = 0.57). The results are summarized in Table 1.

**Table 1:** Variability of olfactory furrow depth and anterior-posterior length of the cribriform plates between genders

	Right side			Left side		
	Male	Female	p-value	Male	Female	p-value
	Keros classification					
Depth (median IQR)	5.7 (1.7)	6.0 (2.0)	0.68	6.2 (1.7)	6.3 (1.8)	0.57
	Yenigun classification					
Length (median IQR)	11.3 (3.6)	11.1 (3.6)	0.32	11.6 (4.0)	11.3 (4.1)	0.28

## Discussion

To our knowledge, our study is the first of its kind to assess the anatomical variation of olfactory fossa in the Pakistani population through Keros and Yenigun classification by CT scan of PNS. When assessing patients before endoscopic sinus surgery (ESS), the CT examination of the nose and paranasal sinus is crucial. Anatomical areas and several disorders, including sinus and nasal anomalies, have greatly benefited from the use of CT scans. Hence, a patient's preoperative assessment for ESS heavily relies on a CT scan of the nose and paranasal sinus [11].

While the likelihood of problems is decreased by anatomic knowledge and expertise, ESS has been linked to a number of troublesome symptoms; mild consequences include infection, hemorrhage, crusting, synechiae, ostial stenosis, and disease recurrence [12]. and serious consequences, including damage to the extraocular muscles, rhinorrhea in the CSF fluid, cerebral injury, and orbital erosion [13]. Compared to the other Keros types, the cribriform plate of the ethmoid skull base, which makes up a significant portion of the roof, is not as protected in type 3. Consequently, type 3 individuals are more vulnerable to iatrogenic side effects [14].

Regarding Keros classification, Keros type II was found to be the most prevalent in our study, followed by type III and type I. In line with this, subsequent studies also find type II to be the most prevalent ​[15-17]. Jang et al. had type II as the most prevalent one, with type I as the second most common category [18].​ Basak et al. found the type II category as the most prevalent one in children [​17]. There were a few studies with exceptions, with type I and type III as the more prevalent categories [19].

When we stratified by gender, type I was more prevalent in females than males, whereas type II and type III were more common in males than females. Type 2 Keros was most common in both males and females. Our results were in accordance with other studies [20,21]. Lakhani et al. and Kaplanoglu et al. found no notable distinctions between the various age groups [22,23]. There is a great deal of diversity in the frequency of type I Keros. Type I was shown to be the least on both sides in females only by Adeel et al., whereas type I was found to be the least on both sides in men only by Kaplanoglu et al. [22,24]. Yet, our research shows that type I Keros is less common in both genders and on both sides. Further studies are required to understand the age-related morphometric changes.

By attempting to locate the ethmoid sulcus in the axial section, the Yenigun classification calculates the anticipated level of the AEA. The likelihood that the AEA will run inside the sinus increases with the anterior-posterior length of the cribriform plate. According to the classification, Yenigun had 53.3% classified as type II (11-16 mm), 41.8% as type I (6-10 mm), and 4.9% as type III (16-20 mm), making it the least common [25]. A study was done in Mexico, and they found that 72.1% of their patients had anterior-posterior length classified into type I, and 24.2% and 3.8% of type II and type III, respectively [26]. A Turkish study mirrored our findings, with type II being the most prevalent, followed by type I and type III [27]. Our results remained consistent with Yenigun et al. and Kolak et al. [25,27]. Muñoz-Leija et al. observed significant gender disparities in Yenigun classifications, specifically between types I and II, with type I being more common [26].

One significant drawback of our study is that the information was gathered from a single tertiary care facility, which limits its generalizability to the larger population. Secondly, the measurement results were analyzed based on values obtained by a single reviewer. Since the intraobserver agreement was not assessed, potential human error may have impacted the findings. Although the reviewer performed duplicate measurements and used the average value for statistical analysis, the small sample size enhances the relevance of this limitation.

## Conclusions

This study's findings, based on CT scan PNS in coronal and axial views for Keros and Yenigun classifications, respectively, provide insight into the distribution of nasal cavity types. Notably, the Keros classification revealed that type 2 was the most prevalent, accounting for 43% of cases, followed by type 3 at 31% and type 1 at 26%. Similarly, the Yenigun classification showed a clear predominance of type 2, making up 51% of cases, with type 1 and type 3 accounting for 29% and 20%, respectively. Interestingly, type 2 Keros emerged as the most common classification across both male and female populations. In the literature, type 2 was the most dominant type in both the Keros and Yenigun classifications. The reason for this seems to be variations in genes, races, and anatomical structures of PNS. Comparing the 3 types of Keros, type 3 is the most dangerous type in which the surgeon can rupture the AEA and enter the skull base. Type 2 is the most commonly encountered, and type 1 is the safest type.

An accurate understanding of ethmoidal roof anatomy is crucial for safe and effective surgical outcomes. Larger-scale studies and meta-analyses are essential to elucidate population-specific variations, guiding optimized preoperative planning.
